# Ileocecal Duplication Cyst Presenting As Bowel Obstruction in an Adult

**DOI:** 10.7759/cureus.103994

**Published:** 2026-02-20

**Authors:** Jessie S Frank, Nanami L Miyazaki, Matthew Ng

**Affiliations:** 1 Department of Surgery, George Washington University Hospital, Washington, USA; 2 Department of Surgery, George Washington University School of Medicine and Health Sciences, Washington, USA

**Keywords:** alimentary tract duplication, duplication cyst in adult, enteric duplication cyst, ileocecal valve duplication cyst, incidental neuroendocrine tumor

## Abstract

Enteric duplication cysts are rare congenital malformations of the gastrointestinal tract, most commonly diagnosed within the first two years of life. In this report, we describe the case of a 56-year-old man who presented to the emergency department with abdominal pain and obstipation. A computed tomography scan revealed a cystic mass at the ileocecal transition point, which was concerning for malignancy. A laparoscopic right hemicolectomy was performed, and the pathology of the specimen revealed an enteric duplication cyst as well as six incidental neuroendocrine tumors. Despite their rare occurrence in adults, enteric duplication cysts should be included in the differential diagnosis of adults presenting with obstructive symptoms, and surgical resection should be strongly considered.

## Introduction

Enteric duplication cysts are rare anomalies of the gastrointestinal tract, with a reported incidence of one in 4,500 births [1]. They may occur anywhere along the alimentary tract but are most commonly found in the small bowel, particularly in the ileum. Duplication cysts are characterized by their close proximity to the gastrointestinal tract, a corresponding epithelial lining, and a smooth muscle layer. They often have a shared blood supply with a nearby region of the alimentary tract, and may or may not communicate with the tract lumen [2,3]. Duplications may be cystic (80%) or tubular (20%) in shape, with tubular duplications more often communicating with the lumen [2,3]. Approximately 20-50% have been reported to contain ectopic gastric mucosa [2,3]. They are characterized as benign lesions; however, due to the potential for malignant transformation and the likelihood of delayed complications, surgery is generally recommended.

Over 80% of enteric duplications are symptomatic within the first two years of life; however, a small proportion go undiagnosed in childhood and are known as “silent” cysts [3]. Enteric duplication cysts are quite rare in the adult population and are often identified either incidentally or upon the onset of complications such as hemorrhage, volvulus, obstruction, perforation, or malignancy. This report describes the case of a 56-year-old man who presented with an acute abdomen and was found to have a duplication cyst of the ileocecal valve as well as six incidental neuroendocrine tumors.

## Case presentation

A 56-year-old man with a history of HIV (undetectable viral load), lymphoma (treated with right inguinal lymphadenectomy and chemotherapy), alcohol use disorder, open left inguinal hernia repair, and an unremarkable colonoscopy three years prior presented to the emergency department with two days of obstipation, abdominal pain, nausea, and nonbloody, nonbilious emesis. Family history was notable for colorectal cancer in his mother, diagnosed in her 80s.

The patient was afebrile and hemodynamically stable on presentation. On physical examination, he appeared pale and diaphoretic. His abdomen was distended, tender to palpation in the lower abdomen and epigastrium, but without rebound tenderness or guarding. His laboratory work-up was largely unremarkable; though he had a white blood cell count nearing the upper limit of the normal range at 10.68 k/mcL and a slightly elevated neutrophil count at 8.56 k/mcL (80%), his overall complete blood count and remaining laboratory values (as reported in Table 1) were without clear indices of severe infection.

**Table 1:** Patient laboratory values on presentation and normal reference range WBC: white blood cell; RBC: red blood cell; Hgb: hemoglobin; Hct: hematocrit; MCV: mean corpuscular volume; MCH: mean corpuscular hemoglobin; MCHC: mean corpuscular hemoglobin concentration; RDW-CV: red cell distribution width; MPV: mean platelet volume; NRBC: nucleated red blood cell; AGAP: anion gap; BUN: blood urea nitrogen; A/G: albumin/globulin; ALP: alkaline phosphatase; AST: aspartate aminotransferase; ALT: alanine aminotransferase; eGFR: estimated glomerular filtration rate

Test	Patient result	Reference range
Complete blood count		
WBC	10.68 × 10^3^/mcL	4.8-10.8 × 10^3^/mcL
RBC	4.63 × 10^6^/mcL	4.7-6.1 × 10^6^/mcL
Hgb	15.3 g/dL	14.0-18.0 g/dL
Hct	44.1%	42.0-52.0%
MCV	95.2 fL	80.0-100.0 fL
MCH	33.0 pg	25.4-34.6 pg
MCHC	34.7 g/dL	33.0-37.0 g/dL
RDW-CV	12.1%	11.5-14.5%
Platelet	296 × 10^3^/mcL	130-400 × 10^3^/mcL
MPV	9.9 fL	7.2-11.1 fL
NRBC (%)	0%	0%
NRBC	0	0
Neutrophils (%)	80%	40-65%
Lymphocytes (%)	14%	21-44%
Monocytes (%)	5%	4-9%
Eosinophils (%)	0%	0-5%
Basophils (%)	0%	0-2%
Immature granulocytes (%)	0.3%	0.1-0.3%
Neutrophils	8.56 × 10^3^/mcL	1.80-7.00 × 10^3^/mcL
Lymphocytes	1.52 × 10^3^/mcL	1.00-4.80 × 10^3^/mcL
Monocytes	0.54 × 10^3^/mcL	0.20-1.00 × 10^3^/mcL
Eosinophils	0.00 × 10^3^/mcL	0.00-0.65 × 10^3^/mcL
Basophils	0.03 × 10^3^/mcL	0.00-0.20 × 10^3^/mcL
Immature granulocytes	0.03 × 10^3^/mcL	0.01-0.03 × 10^3^/mcL
Comprehensive metabolic panel		
Glucose level	184 mg/dL	75-110 mg/dL
Sodium	140 mmol/L	135-145 mmol/L
Potassium	4.1 mmol/L	3.5-5.0 mmol/L
Chloride	102 mmol/L	95-105 mmol/L
CO_2_	21.0 mmol/L	22-30 mmol/L
AGAP	17 mmol/L	≤11 mmol/L
BUN	16 mg/dL	10-20 mg/dL
Creatinine	0.9 mg/dL	0.8-1.5 mg/dL
BUN/creatinine ratio	18	-
Calcium	10.6 mg/dL	8.5-10.5 mg/dL
Albumin	5.3 g/dL	3.5-5.0 g/dL
Total protein	8.9 g/dL	6.0-8.0 g/dL
A/G ratio	1.47	-
Total bilirubin	1.4 mg/dL	0.2-1.3 mg/dL
ALP	59 units/L	40-125 units/L
AST	30 units/L	10-45 units/L
ALT	22 units/L	10-45 units/L
eGFR	110 mL/minute/1.73 m^2^	≥90 mL/minute/1.73 m^2^
Urinalysis		
Color	Yellow	Yellow
Clarity	Clear	Clear
Urine specific gravity	1.020	1.003-1.035
pH	5.0	5.0-8.0
Leukocyte esterase	Trace	Negative
Nitrite	Negative	Negative
Protein	Negative	Negative
Urine glucose	50 mg/dL	Negative
Urine ketones	20 mg/dL	Negative
Urobilinogen	Normal	Normal
Bilirubin	Negative	Negative
Blood	Negative	Negative
WBC	23	0-4/HPF
RBC	6	0-2/HPF
Squamous epithelial cells	2	0-4/HPF
Mucous	Rare	None
Amorphous	Rare	0-moderate

**Figure 1:**
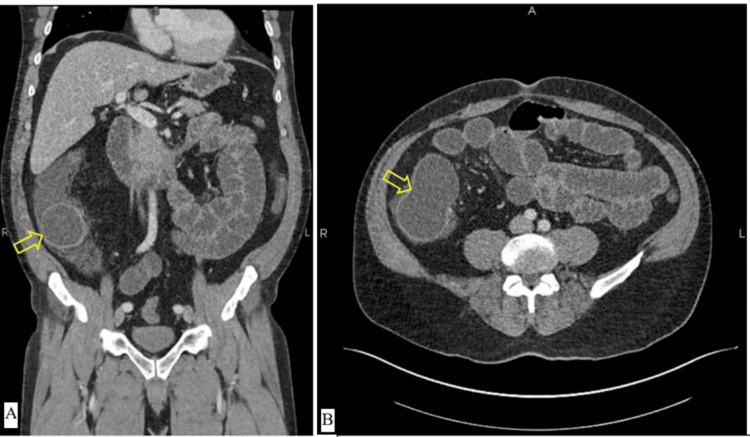
Preoperative computed tomography imaging with intravenous contrast of the abdomen and pelvis (A: coronal; B: axial) showing a 9.3 x 4.9 x 5.4 cm lobulated cystic mass at the ileocecal transition point, indicated by yellow arrows

Computed tomography of the abdomen and pelvis showed multiple fluid-filled loops of small bowel and a thin-walled, bilobed cystic mass measuring 9.3 x 4.9 x 5.4 cm at the ileocecal valve with an associated transition point. The terminal ileum was visualized posterior and inferior to the cystic mass (Figure 1). There was no evidence of bowel ischemia. Given the computed tomography findings, the patient’s history of lymphoma, and his family history of colorectal cancer, there was heightened suspicion for malignancy. He was admitted for nasogastric tube decompression, fluid resuscitation, and diagnostic laparoscopy.

**Figure 2:**
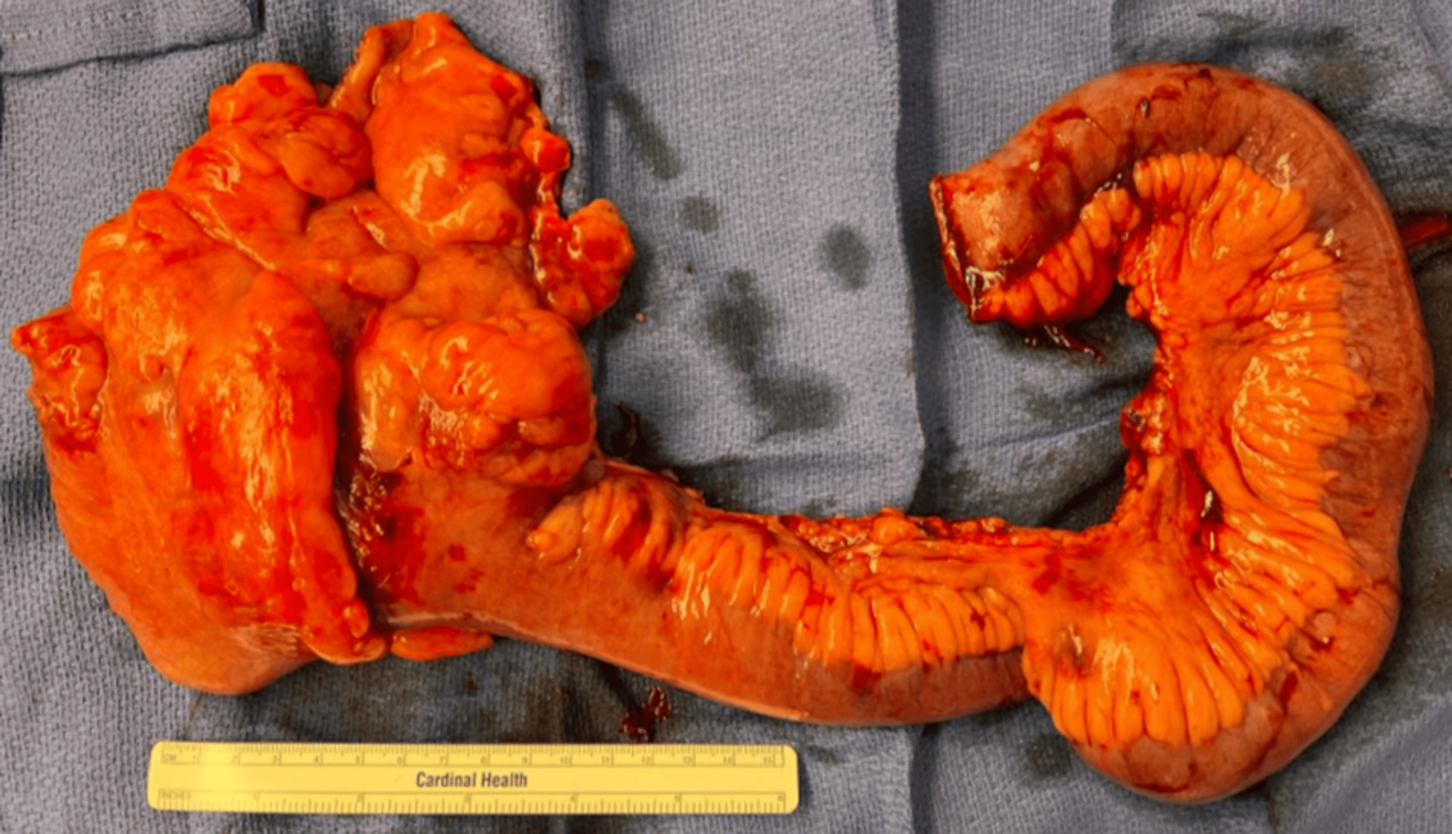
Gross specimen of right hemicolectomy with a cystic mass

In the operating room, a soft, cystic-appearing mass was noted on the antimesenteric surface at the junction of the terminal ileum and the cecum. The appendix and the cecum itself both appeared normal. The small bowel was run in a retrograde direction from the terminal ileum to the ligament of Treitz. The small bowel was noted to be fairly thin-walled, and there was evidence of a resolving small bowel obstruction. No discrete transition point was identified. Given the location of the mass, its adherence to the bowel wall, and our increased concern for malignancy, a formal right hemicolectomy with a side-to-side functional end-to-end isoperistaltic anastomosis was performed. The gross specimen was sent to pathology (Figure 2).

The patient did well postoperatively. He had a return of bowel function on postoperative day 4. On postoperative day 6, he was started on a 10-day course of antibiotics for cellulitis at his extraction site. He was medically stable for discharge on postoperative day 7 with the remainder of his antibiotic course and a scheduled outpatient follow-up appointment six days later. By the time of his first office visit, he had no further evidence of surgical site infection, he was tolerating a regular diet, and he endorsed full resolution of his presenting symptoms.

**Figure 3:**
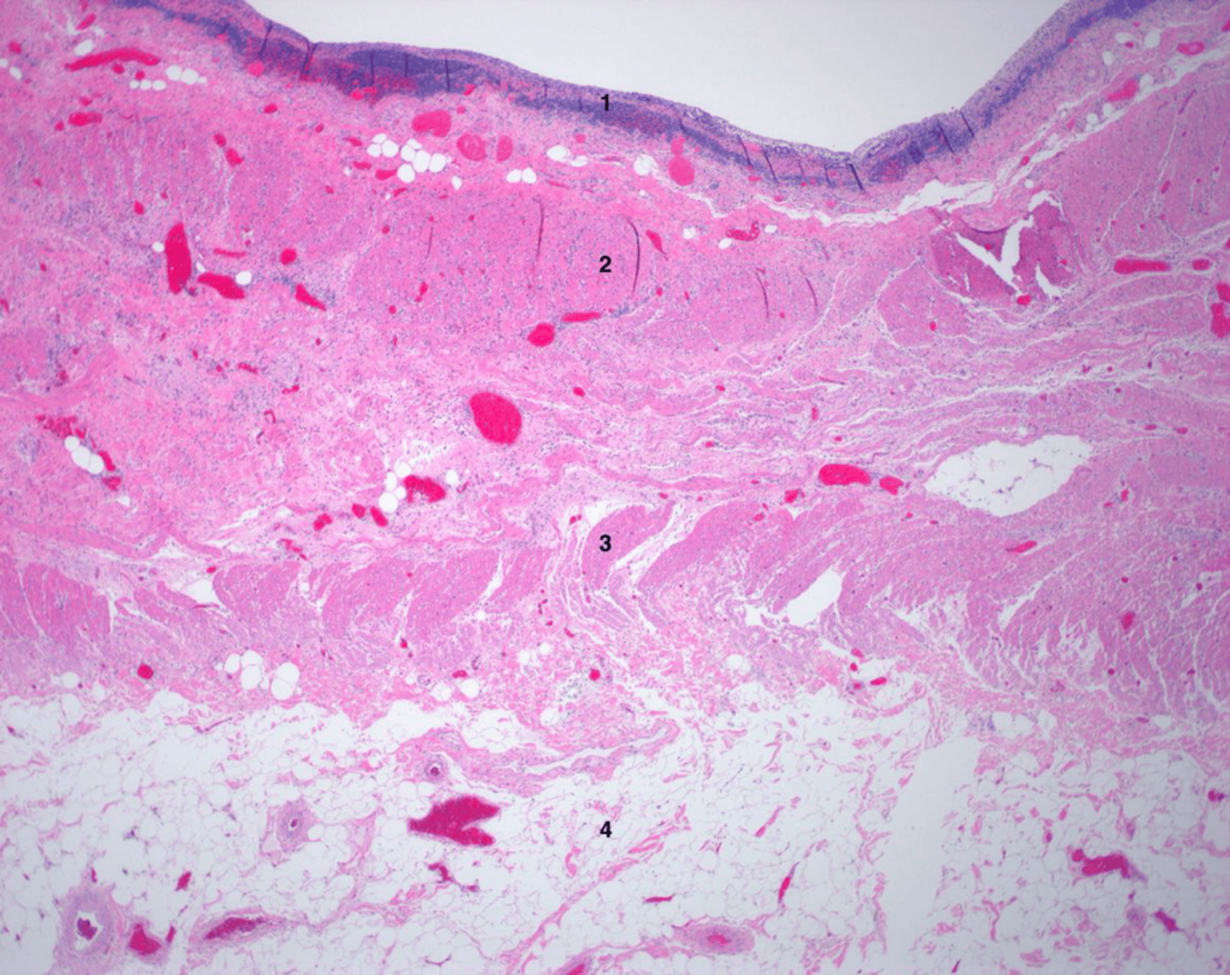
Histology of the duplication cyst and ileocecal valve, with layers as follows: 1) cyst mucosa, 2) muscularis propria of the cyst, 3) muscularis propria of the ileocecal valve, and 4) submucosa of the ileocecal valve containing abundant adipose tissue

**Figure 4:**
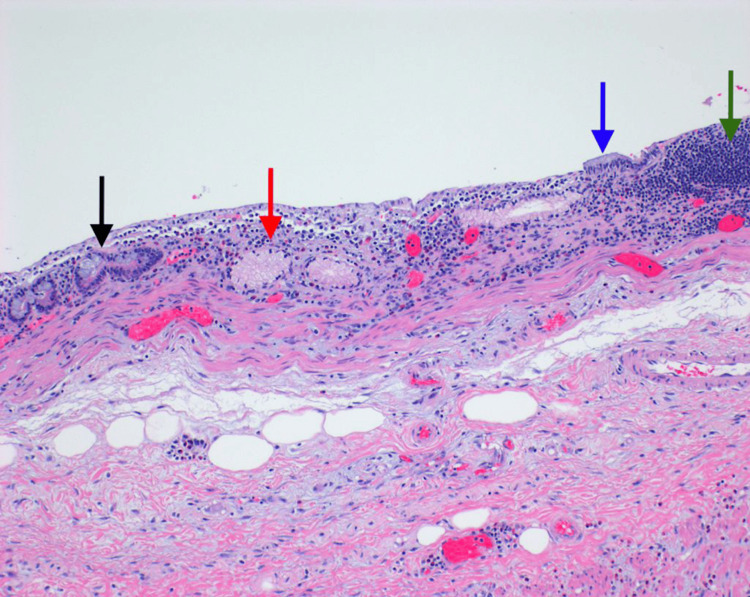
Histological image (10x) of the duplication cyst mucosa. Black arrow: intestinal crypts. Red arrow: pyloric gland metaplasia, which was found extensively in the mucosal lining of the cyst. Blue arrow: residual surface epithelial lining, which is columnar/intestinal in nature. Green arrow: lymphoid aggregates that were also extensive throughout the cyst mucosal lining

Final pathology of the surgical specimen demonstrated an ovoid, intact duplication cyst filled with thin, clear fluid located at and noted to be obstructing the ileocecal valve. The cyst displayed a lining of atrophic mucosa with lymphoid aggregates and pyloric gland metaplasia, as well as a muscle layer continuous with the bowel wall (Figures 3, 4). Final pathology also revealed incidental findings of one tubular adenoma of the cecum and six well-differentiated neuroendocrine tumors of the ileum, each with negative margins. All 23 resected lymph nodes were benign. The neuroendocrine tumors were found to be Grade G1 and American Joint Committee on Cancer pathologic stage mT1N0MX.

For further evaluation and management of the incidentally found neuroendocrine tumors, he was set up for a positron-emission tomography/computed tomography DOTATATE scan and referred to oncology. Imaging showed no evidence of hypermetabolic lymph nodes but foci of hypermetabolic activity within the prostate. He was found to have an elevated prostate-specific antigen and underwent magnetic resonance imaging of the pelvis, which identified three prostatic findings compatible with Prostate Imaging-Reporting and Data System 3 lesions. He opted for active surveillance management with planned repeat blood work and possible biopsy in six months.

## Discussion

The presentation of enteric duplication cysts can vary based on their anatomical location. Foregut duplications may present with dysphagia and epigastric pain; midgut duplications may present with a palpable mass, intussusception, or volvulus; and hindgut duplications may present with a palpable mass or obstruction [2]. Regardless of location, common presenting symptoms also include abdominal pain, nausea, vomiting, and upper or lower gastrointestinal bleeding [1,2]. Other complications of duplication cysts include infection, gastrointestinal perforation from ectopic mucosal secretions, and, rarely, malignant transformation [2,4]. The most common malignancy identified is adenocarcinoma, followed by squamous cell carcinoma and then neuroendocrine tumors [3,5,6]. Colonic duplication cysts have the highest rate of malignant transformation [5].

Due to their variable location and presenting symptoms, enteric duplication cysts are often part of a very broad list of differential diagnoses, to include mesenteric, omental, choledochal, or ovarian cysts; Meckel’s diverticula; congenital segmental intestinal dilatation; presacral masses; other intrathoracic cysts; or, as in the present case, either primary malignancy or a recurrence of our patient’s lymphoma [7]. Bergeron and Gervais report a case of an enteric duplication cyst, initially assessed as a hydrosalpinx and later thought to be an appendiceal mucocele, before a final diagnosis of duplication was made [8]. Weitman et al. report a case of an enteric duplication cyst, which was initially thought to be a mucinous cystic neoplasm of the pancreas [9]. Definitive preoperative diagnosis of an enteric duplication cyst is difficult to establish, particularly in the adult population.

The definitive treatment of an enteric duplication cyst is complete surgical resection [1,10]. In cases of completely isolated duplication cysts, it is possible to safely resect the cyst alone [11-13]. However, cysts that are attached to the gastrointestinal tract share a wall and vascular supply and may require removal of the surrounding gastrointestinal tissue as well [1]. In our case, the duplication cyst shared its muscular wall with the ileocecal valve, necessitating a wider resection.

For this obstructive lesion involving the cecum and terminal ileum, with a high preoperative probability of malignancy in the context of the patient's HIV history and prior lymphoma, we elected an oncologic right hemicolectomy rather than a more limited segmental or ileocecal resection. This decision was guided by several key factors: the lesion caused complete obstruction, preventing endoscopic traversal, biopsy, or exclusion of malignancy; the patient's risk factors significantly elevated concern for an aggressive malignant process (recurrent lymphoma or de novo adenocarcinoma), and an oncologic resection was required to ensure adequate regional lymphadenectomy for accurate staging and potential cure. A limited resection would risk insufficient nodal sampling, potentially necessitating adjuvant chemotherapy if malignancy was confirmed, whereas a formal right hemicolectomy offered the best opportunity for R0 resection and long-term disease-free survival without systemic therapy in the event of node-negative disease. This approach aligns with standard oncologic principles for right-sided lesions with high malignant potential.

The current case is notable for several reasons. First, it is one of only a handful of reports of an ileocecal valve duplication cyst presenting in an adult [14-16]. This is perhaps because an ileocecal valve duplication may be more likely to present with a complication early in life, such as intussusception or obstruction. The second reason this case is notable is simply that 56 years of age is advanced for the presentation of a complication of a duplication cyst, regardless of its location. Most reports of enteric duplication cysts in adults describe an earlier presenting age.

Finally, this case is notable due to the finding of six incidental neuroendocrine tumors. To our knowledge, this is only the third reported case of incidentally found neuroendocrine tumors with an enteric duplication cyst. Bellanova et al. reported the case of a 39-year-old woman who presented with abdominal pain, underwent surgical excision of two duplication cysts (ileum and transverse colon), and was incidentally found to have a neuroendocrine tumor of her appendix [7]. Siragusa et al. reported the case of a 26-year-old woman who presented with dysmenorrhea, underwent surgical excision of an isolated ileal duplication cyst, and was also incidentally found to have an appendiceal neuroendocrine tumor [17]. This patient subsequently underwent a laparoscopic right hemicolectomy.

Neuroendocrine tumors have a reported annual incidence of 2.5-5 in 100,000 people and are most commonly located in the small intestine [18]. According to the 2024 National Comprehensive Cancer Network Guidelines on neuroendocrine tumors, surgical resection of jejunal, ileal, or colonic neuroendocrine tumors along with regional lymphadenectomy is recommended for nonmetastatic disease [19]. A 10-year survival rate is reported at 100% for Stage I and II disease [20]. In the current case, adequate resection and lymphadenectomy were obtained due to the high preoperative suspicion for other malignancies.

## Conclusions

Enteric duplication cysts are rare in adults. They should be considered in the differential diagnosis of patients presenting with vague abdominal symptoms or acute abdomen alike. When an enteric duplication cyst is suspected, surgical resection is encouraged to reduce the risk of further complication, including malignant degeneration.
